# Mass barium carbonate poisoning with fatal outcome, lessons learned: a case series

**DOI:** 10.4076/1757-1626-2-9069

**Published:** 2009-09-01

**Authors:** Aniruddha Ghose, Abdullah Abu Sayeed, Amir Hossain, Ridwanur Rahman, Abul Faiz, Gofranul Haque

**Affiliations:** 1 Department of Medicine, Chittagong Medical College, Chittagong, 4000, Bangladesh; 2 Department of Medicine, Shaheed Suhrawardy Medical College, Dhaka, 1207, Bangladesh; 3 Department of Medicine, Sir Salimullah Medical College, Dhaka, 1000, Bangladesh

## Abstract

**Introduction:**

Barium, a heavy divalent alkaline metal, has long been known to cause human toxicity. The common mode is accidental ingestion and the common compound is Barium carbonate. Here we report an incident of food poisoning in 27 law enforcement personnel with rapidly developing sequelae and a high mortality due to ingestion of Barium carbonate contaminated flour.

**Case presentation:**

One midnight, 27 adult males were rushed to emergency department of Chittagong Medical College Hospital with abdominal pain, vomiting, loose motion, cramps and generalized paraesthesia. The ailment started 1-2 hours after Iftar (evening meal to break day long fast during Ramadan) which included fried vegetables coated with a flour paste. On admission, twenty of them were restless, agitated. 22 reported weakness of limbs and were unable to walk. 10 had hypotension. 22 had rapid and shallow respiration. 5 had carpopedal spasm. Different grades of limb weakness were noted with loss of tendon jerks. Ten (N12) patients had hypokalaemia, three had hypoglycaemia, 4 patients had high creatine kinase. ECG showed flat ST with U waves in 4 patients. Potassium containing intravenous fluid and Oxygen was administered. Due to limited availability of mechanical ventilators patients were put on artificial respiration using Ambu bag; manually maintained by doctors, paramedics and attendants. Four patients were transferred to another hospital for mechanical ventilation. A total of 12 patients died over next 16 hours, 4 within 3 hrs. Other patients gradually improved. Chemical analysis of the vomitus, blood and flour used for preparation of meal revealed the presence of Barium. It was assumed that the flour was contaminated with the similar looking Barium carbonate powder which was kept in the kitchen as a rodenticide.

**Conclusion:**

This event exemplifies the weakness of usual health care facility in resource poor settings to cope with this kind of massive poisoning event. The multiple reported incidences of accidental barium poisoning due to unintentional mixing with food signifies the fact that the use and availability of barium carbonate should be restricted. We hope to draw attention to this relatively uncommon poisoning and to the need for development of poison information centre in resource poor countries.

## Introduction

Barium, a heavy divalent alkaline metal has long been known to cause human toxicity. Currently approximately 40 of these salts are being used in industries [1]. Insoluble Ba sulphate is used as radiographic contrast medium. Carbonate, hydroxide and chloride forms of barium are used in pesticides. Ba carbonate is also used for glazing pottery while barium sulphide is used in depilators for external application [2].

Stomach acid converts the Ba carbonate to Ba chloride and intestinal absorption is similar to calcium and excretion is mainly faecal. Peak serum level occurs 2 hours after ingestion with an elimination half life of 3.6 days. The systemic effects of Barium are linked with two modes of action: direct muscular stimulation (skeletal, cardiac, and smooth), and hypokalaemia [3,4]. The latter effect is associated with the ability of the barium ion to block potassium (K+) channels and interfere with passive K+ diffusion [4,5]. All water and acid soluble Ba salts are poisonous. Case reports implicating several compounds of Ba (eg. Carbonate, chloride) have been reported in literature. The common mode is accidental ingestion and the common compound is Ba carbonate. Accidental mixing of Ba carbonate with food and poisoning out of it has been reported as early as in the 2^nd^ world war [6]. Ba Carbonate exposure has not been previously reported In Bangladesh. The use of Ba carbonate as rodenticide and subsequent poisoning is relatively unknown to the medical community in Bangladesh. We report here an incident of food poisoning in 27 law enforcement personnel with rapidly developing sequelae and a high mortality due to the ingestion of Ba carbonate contaminated flour. We hope to draw attention to this relatively unknown and uncommon presentation and to the need to develop a poison information centre in resource poor countries.

### Chronology and description of cases

On the evening of 17th November 2001, at around 6 pm, 27 armed police personnel in a camp in a remote area of Chittagong Hill Tracts simultaneously developed abdominal pain and vomiting followed by loose motions all within 30–60 minutes of each other. Their symptoms progressed rapidly, and subsequently they developed cramps, pains, generalized paresthesia, most marked in the limbs. At around 10 pm they were taken to a nearby hospital, where they were given oral rehydration and IV fluids and were referred to Chittagong Medical College Hospital. They arrived at the emergency between 11:45 pm to 12:30 am and were quickly transferred to medicine ward. All 27 patients were interviewed and examined. It was found that they all had taken Iftar (the evening meal to break day long fast during the month of Ramadan) consisting of traditional food items which included beans, pulse, and fried vegetables coated with a flour paste. The ailment started 1-2 hours after the meal.

On admission all of them presented with features of acute gastroenteritis. Twenty of them were restless and agitated. Raised blood pressure (SBP 150-170, DBP 90-110) was noted in 5 patients, 10 were found to have BP less than 100/70 mm Hg. Rapid and shallow respiration were found in 22 patients. Twenty two patients reported weakness of all four limbs and were unable to move or walk. Five patients had carpopedal spasm. Different grades of limb weakness were noted with loss of tendon jerks. Gastric aspiration, stool, and blood samples were collected from 24 patients. ECG was done in 12 patients. Serum electrolytes, CPK and creatinine were measured in 12 patients. Ten patients had hypokalaemia (K^+^< 3.5) ranging from 1.87 to 3.05. Three were hypoglycaemic. Four patients had high CPK (cut off value 190 u/l). ECGs showed flat ST segments with U waves in 4 patients, one patient had ECG features resembling ischaemia, three had left ventricular hypertrophy.

Intravenous fluid rich in K^+^ were given to all patients. Oxygen supplementation with nasal prongs was established. Regular monitoring of vital signs, fluid balance was arranged. Seven patients rapidly deteriorated. Four of them died within 3 hrs of admission due to respiratory paralysis. Due to limited availability of mechanical ventilators patients were put on artificial respiration using Ambu bags; manually maintained by doctors, paramedics and attendants. Four patients were transferred to Combined Army Medical Hospital for mechanical ventilation. In total 12 patients died over next 16 hours; all apparently due to respiratory failure. Other patients gradually improved over time and discharged subsequently. Their recovery was uneventful.

Chemical analysis of the vomitus and blood samples from the victims revealed presence of Barium. Barium was also detected in the sample of flour used for preparation of meal. It was assumed that the flour was contaminated with the similar looking Ba carbonate powder which was kept in the kitchen as a rodenticide.

## Discussion

The patients in this event passed though several stages. Initially all developed gastroenteritis, subsequently nervous system was affected as evident by tingling, numbness and limb weakness due to neuromuscular paralysis and later on some of them developed respiratory muscle paralysis. Suspicion of food poisoning was made from the clustered nature of these cases and the temporal relationship with meal. With the rapid onset of features of gastroenteritis and neuromuscular paralysis the differential diagnoses in this situation were limited. Ciguetra and paralytic shellfish were excluded from the list of consumed food items. The other closely related conditions were gastroenteritis with hypokalaemic paralysis and Botulism, but the prominent sensory symptoms could not be explained by any of these. Botulism usually takes a longer time to develop [1].

Similar incidences of mass food poisoning due to Ba Carbonate have been reported previously. In 1945 in a British army regiment Ba carbonate was identified as the agent causing poisoning in 89 soldiers. Two outbreaks of Ba Carbonate poisoning happened in Israel in a large number of people in different villages [7]. Johnson HC reported seven cases of Ba Carbonate poisoning occurring in one family [1]. In all of these cases the common factor appeared to be the accidental mixing of Ba Carbonate with foods. Ba carbonate powder has a similar appearance to flour and flour was used to prepare these food items. The clinical features of our patients also resemble those in previous reports. In all these events, features suggesting gastroenteritis were the presenting complaints with patients subsequently developing muscle weakness and cardiac rhythm abnormalities. One striking difference is that we detected no rhythm abnormality in our patients.

In this series 12 patients died. This is a much higher mortality than in any other previously reported case series and is surprising given the greater expected physical reserve of armed force personnel compared to general population. Another feature unique to this group is that they were all fasted for about 12 hours prior to poisoning. The long fast and fluid depletion might have accelerated the absorption of Ba carbonate and a larger than normal amount of food may have been consumed in one sitting following the fast. This may explain the rapid involvement of respiratory muscles leading to respiratory failure with hypokalaemia contributing. Due to limited resource we were unable to perform all the necessary investigations in all patients. For those who survived (N15), several factors might have contributed, e.g. less amount ingested, better intravenous potassium replacement and effective respiratory support.

A high index of suspicion along with correlation of the clinical events with the circumstantial evidence is very important in diagnosis and management of uncommon poisonings. After the initial gastric lavage, further management is largely supportive [8]. Endotracheal intubation and mechanical ventilation is indicated if respiratory failure intervenes. Sodium sulphate given orally has been claimed to reduce absorption by forming an insoluble, nontoxic Ba sulphate. Intravenous magnesium and potassium chloride are used to correct hypomagnesaemia and hypokalaemia, respectively. Renal replacement therapy (intermittent haemodialysis [9]-[11] or continuous venovenous haemodiafiltration [3] shortens the half-life of barium and is reported to be effective in severe barium poisoning and should be considered early in the management.

Another very important aspect of this event is that it signifies the need for further development of traditional health care facilities to cope with this kind of massive poisoning event. The sheer number of patients presenting in an acute emergency condition in an ordinary medicine ward was not something easily dealt with. The on duty doctors were out numbered. We tackled the situation by summoning doctors and consultants from other units in the middle of the night. Due to the limited availability of respiratory support, some patients had to be transferred to another hospital and others were managed by manual ventilation. If we had more equipped ICU or HDU facilities the fatalities would probably have been less. During the initial management of these cases we were at a loss regarding the possible differential diagnoses and necessary laboratory investigations. The importance of a poison information centre to tackle this kind of situation has been elaborately highlighted by Deng JF et al [12]. Should there be an established poison information centre we could have used the resources and the management would have been better with a more favorable outcome.

## Conclusion

The multiple reported incidents of accidental Ba poisoning due to unintentional mixing with food signifies the fact that the use and availability of Ba Carbonate should be restricted. The proposal of colouring the powder of Ba Carbonate so that it can not be mistaken as flour demands serious appreciation from the concerned authority. Medical professionals should also be aware of the possibility of such accidents and be cautious in suspicious cases. The essential role of a resourceful poison information centre in aiding the diagnosis, assisting with laboratory confirmation and management of such an incident can not be over stated.

## Abbreviations

BA: barium; CPK: creatine kinase; DBP: diastolic blood pressure; SBP: systolic blood pressure.

## Consent

Written informed consent was obtained from the patients for publication of this case report. A copy of the written consent is available for review by the Editor-in-Chief of this journal.

## Competing interests

The authors declare that they have no competing interests.

## Authors' contributions

AG attended the patients, involved in collection of data, literature search, and manuscript writing. AAS involved in searching literature and manuscript editing. AH performed data collection, and manuscript editing. MRR, MAF and MGH were the consultant physicians attending the patients, and involved in editing the manuscript.
